# Changing times: trends in risk classification, tumor upstaging, and positive surgical margins after radical prostatectomy - results from a contemporary National Cancer Database study

**DOI:** 10.1007/s00345-024-05262-0

**Published:** 2024-09-30

**Authors:** Pedro F. S. Freitas, Ruben Blachman-Braun, Nachiketh Soodana-Prakash, Adam D. Williams, Chad R. Ritch, Sanoj Punnen, Mark L. Gonzalgo, Dipen Parekh, Bruno Nahar

**Affiliations:** Desai Sethi Urology Institute, University of Miami Miller School of Medicine, 1120 NW 14th St. Miami, Miami, FL 33136 USA

**Keywords:** Prostatic neoplasms, Prostatectomy, Database, Margins of excision

## Abstract

**Purpose:**

Recent advancements in screening, prostate MRI, robotic surgery, and active surveillance have influenced the profile of patients undergoing radical prostatectomy (RP). We sought to examine their impact on trends in clinicodemographic, risk classification, and adverse pathology in men undergoing surgery.

**Methods:**

We queried the National Cancer Database for clinicodemographic, risk group, and pathology data in men undergoing upfront RP between 2006 and 2020. Patients were categorized by NCCN risk groups, and trends were assessed among 2006–2010, 2011–2015, and 2016–2020 periods. Endpoints included rates of pT3, positive surgical margins (PSM), pathologic upstaging, and Gleason grade group (GG) upgrading.

**Results:**

610,762 patients were included. There were significant increases in African Americans (9.8–14.1%), comorbidities (2.1–5.2% with Charlson scores > 1), and robot-assisted RP (78–84%). Over the three time periods, high-risk cases increased from 15 to 20 to 27%, and intermediate-risk from 54 to 51 to 60%. Overall rates of pT3 rose from 20 to 38%, and PSM from 20 to 27% (*p* < 0.001). Pathologic upstaging increased in low (6–15%), intermediate (20–33%), and high-risk groups (42–58%) –*p* < 0.001. Gleason upgrading rose in low-risk (45–59%, *p* < 0.001), with slight reductions in the intermediate and high-risk groups.

**Conclusions:**

Recent trends in RP indicate a shift towards more advanced disease, evidenced by increasing rates of pT3, PSM, and pathologic upstaging across all NCCN risk groups. These findings emphasize the need for a careful balance in applying fascia and nerve-sparing techniques to avoid compromising oncological safety.

## Introduction

Prostate Cancer (PCa) has experienced significant changes in both diagnostic and treatment methods over the past fifteen years. Screening practices that initially involved widespread, non-selective methods, then shifted following the US Preventive Services Task Force recommendations for a more selective approach [1], emphasizing a shared decision-making process [2]. On top of policy swings, adherence to guidelines differs in time and across specialties, societies, and providers [3, 4]. Management strategies have undergone notable changes, including the increasing adoption of active surveillance (AS), the introduction of focal therapy, and the transition from open to robot-assisted radical prostatectomy (RP) [5, 6]. As RP has evolved, with reduced morbidity, accumulated surgical experience, and advancements in technology, its use has become more prevalent, particularly among high-risk patients [3, 7].

These transformations may modify the profile of patients undergoing RP. In fact, retrospective series have indicated increasing age, comorbidities, and PCa risk [8–10] among RP candidates. These shifts are not merely of demographic or descriptive significance; they also hold the potential to impact the functional and oncological outcomes experienced by patients. Additionally, the presence of residual or recurrent disease after RP implies a greater need for advanced imaging, multidisciplinary care, and additional treatment such as radiation therapy (RT) and androgen deprivation therapy [8, 11]. This changing scenario is relevant to clinicians who must provide patients with up-to-date data on oncological and functional outcomes of RP and later define the most appropriate surgical strategy.

To our knowledge, this is the first study to address these changes in a large contemporary RP population. We sought to characterize temporal trends in sociodemographic, risk profiles, adverse pathologic features, and the need for additional therapy in men undergoing RP. We hypothesized that RP is increasingly being performed on men with higher-risk disease, potentially influencing tumor upstaging and positive surgical margins rates.

## Materials and methods

### Data source

The National Cancer Database (NCDB) is the largest oncology registry in the United States, capturing over 70% of cancers diagnosed and treated in hospitals annually [12]. It is the product of, and maintained by, a joint initiative between the Commission on Cancer of the American College of Surgeons and the American Cancer Society. Deidentified sociodemographic, clinical, and treatment information is collected from more than 1,500 accredited hospitals by trained personnel on a standard methodology [12].

### Cohort selection

**Fig. 1:**
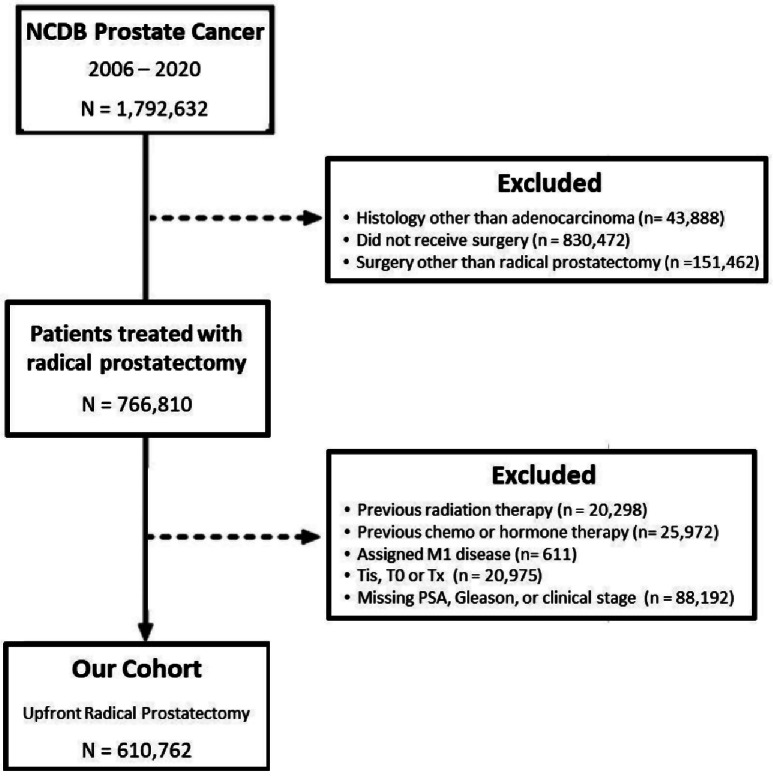
Flow chart detailing patient inclusion and exclusion criteria

Men > 18 years of age diagnosed with prostate adenocarcinoma who underwent radical prostatectomy as their primary treatment between 2006 and 2020 were identified using the International Classification of Diseases for Oncology, 3rd edition (topography code C61.9). This period was intentionally set after the major revision in the Gleason scoring system introduced in 2005 to allow for a more consistent evaluation of temporal trends, thus avoiding possible confusion with earlier versions. Patients who had received previous radiation or hormone therapy, presented with distant metastasis, or had missing data regarding the procedure, risk group determinants (PSA, Gleason, clinical T stage), or surgical margin status were excluded. The study population selection process is detailed in Fig. 1. Selecting a broad time frame and applying few exclusion criteria were deliberate choices to enable a real-world evaluation of radical prostatectomy (RP) utilization across various risk groups and disease scenarios.

### Data extraction and endpoints

We collected sociodemographic data including age, race, ethnicity, Charlson-Deyo comorbidity score, insurance status, and rurality status. Treatment information, including year, facility type (academic or community), and RP approach (robot-assisted vs. other), were extracted. Preoperative clinical risk determinants including Gleason score (GS), PSA levels, and clinical T staging were compiled and patients were classified according to a modified version of the NCCN Guidelines into low risk (cT1c–T2a, GS ≤ 6, and PSA < 10 ng/mL), intermediate risk (cT2b–T2c, GS 7, or PSA 10–20 ng/mL), and high risk (cT3–T4, GS 8–10, or PSA > 20 ng/mL). Finally, the study population was divided into three sequential time periods (2006–2010, 2011–2015, and 2016–2020) to assess temporal trends of the above-mentioned variables.

The endpoints were rates of pathologic T3 (pT3) disease, positive surgical margins, pathologic upstaging, and Gleason upgrading on surgical specimen analysis. Pathologic upstaging was defined as cT1-T2 tumors that were ultimately found to be pT3-T4 (pT3+) upon surgical specimen analysis. Upstaging rates were calculated by dividing the number of upstaged men by all patients with cT1-T2 (i.e. exposed to upstaging as per this definition) in each NCCN risk group. Gleason upgrading was defined as any increase in Grade Group (GG 1–5) – including GG2 to GG3 in intermediate-risk patients – at the surgical specimen compared to pre-operative (biopsy) values. Similarly, upgrading rates were calculated separately for each risk group by dividing the number of upstaged cases by the total of cases in each NCCN category. Temporal trends were evaluated across the different NCCN risk groups by year and across the three time periods described above. As both robotic surgery and post-operative Gleason scores were not significantly captured until 2010, these two variables were exceptionally analyzed considering only the two latest time periods (i.e. 2011–2015 and 2016–2020).

### Statistical analysis

We compared characteristics among the three time periods using an ANOVA test for continuous variables and a Chi-squared test for proportions. Changes in the relative frequency of variables and primary endpoints over time were assessed using the Mantel–Haenszel test for trends. Statistical analysis was performed using SAS v. 9.4 software (SAS Institute, Cary, NC, USA). A P value < 0.05 was considered significant.

## Results

A total of 610,762 patients underwent RP between 2006 and 2020 and met the inclusion criteria. Baseline sociodemographic, clinical, and treatment characteristics are detailed in Table 1. There were no clinically relevant changes in median age across the different eras. We found a statistically significant increase in the frequency of comorbidity scores (2.1–5.2% with Charlson scores > 1) and the use of robot-assisted RP (78–84%).

**Table 1:** Patient cohort characteristics

	All	2006–2010	2011–2015	2016–2020	*p* value
Patients	610,762	202,837 (33.2)	196,126 (32.1)	211,799 (34.7)	
Age, Median (IQR)	62 (57, 67)	61 (56, 66)	62 (56, 66)	63 (58–68)	< 0.001
Race, *n* (%)					
White	507,102 (83.0)	172,747 (85.2)	162,796 (83.0)	171,559 (81.0)	< 0.001
Black	75,945 (12.4)	20,963 (10.3)	25,148 (12.8)	29,834 (14.1)	
Other / Unknown	27,715 (4.6)	9,128 (4.5)	8,181 (4.2)	10,406 (4.0)	
Ethnicity *n* (%)					
Hispanic	26,053 (4.5)	6,966 (3.8)	8,301 (4.3)	10,786 (5.2)	< 0.001
Non-Hispanic	557,950 (95.5)	178,519 (96.2)	182,495 (95.7)	196,936 (94.8)	
Rurality, *n* (%)					
Metro	478,486 (80.3)	157,388 (79.5)	153,186 (80.0)	167,912 (81.4)	< 0.001
Urban	74,968 (12.6)	23,691 (12.0)	24,588 (12.9)	26,679 (12.9)	
Rural	9,862 (1.7)	3,404 (1.7)	3,195 (1.7)	3,263 (1.6)	
Unknown	32,549 (5.4)	13,586 (6.8)	10,440 (5.4)	8,523 (4.1)	
Charlson Index, *n* (%)					
Score = 0	501,512 (82.1)	169,112 (83.4)	159,975 (81.5)	172,425 (81.4)	< 0.001
Score = 1	88,441 (14.5)	29,381 (14.5)	30,724 (15.7)	28,336 (13.4)	
Score > 1	20,809 (3.4)	4,344 (2.1)	5,427 (2.8)	11,038 (5.2)	
Facility Type, *n* (%)					
Academic	259,602 (42.5)	86,793 (42.8)	82,667 (42.2)	90,142 (42.6)	< 0.001
Community	350,620 (57.5)	115,768 (57.2)	113,289 (57.8)	121,563 (57.4)	
NCCN Risk Group, *n* (%)					
Low	142,794 (23.9)	59,774 (30.9)	55,206 (28.4)	27,814 (13.2)	< 0.001
Intermediate	328,874 (55.0)	103,850 (53.7)	99,534 (51.3)	125,490 (59.8)	
High	126,071 (21.1)	29,844 (15.4)	39,472 (20.3)	56,755 (27.0)	
Robot-Assisted, *n* (%)	362,139 (81.3)	N/A	152,818 (77.9)	178,644 (84.4)	< 0.001
Clinical N1 Stage, *n* (%)	1,881 (0.3)	398 (0.2)	562 (0.3)	921 (0.5)	< 0.001
Pathologic T Stage, *n* (%)					
pT2	421,791 (70.7)	154,908 (79.5)	138,519 (71.9)	128,364 (61.3)	< 0.001
pT3	173,190 (29.0)	38,896 (20.0)	53,830 (27.9)	80,434 (38.4)	
pT4	1,892 (0.3)	969 (0.5)	396 (0.2)	527 (0.3)	
Pathologic N1, *n* (%)	20,998 (4.5)	3,502 (2.4)	6,382 (4.4)	11,114 (6.7)	< 0.001
Post-Op Gleason Score					
≤6	79,343 (17.4)	16,555 (32.9)	44,228 (22.6)	18,560 (8.9)	< 0.001
7	314,303 (69.1)	29,190 (58.0)	128,272 (65.7)	156,841 (74.9)	
≥8	61,257 (13.5)	4,572 (9.1)	22,805 (11.7)	33,880 (16.2)	

*p-values were calculated using the Mantel-Haenszel test for trends for categorical variables and ANOVA tests were used for continuous variables

**Fig. 2:**
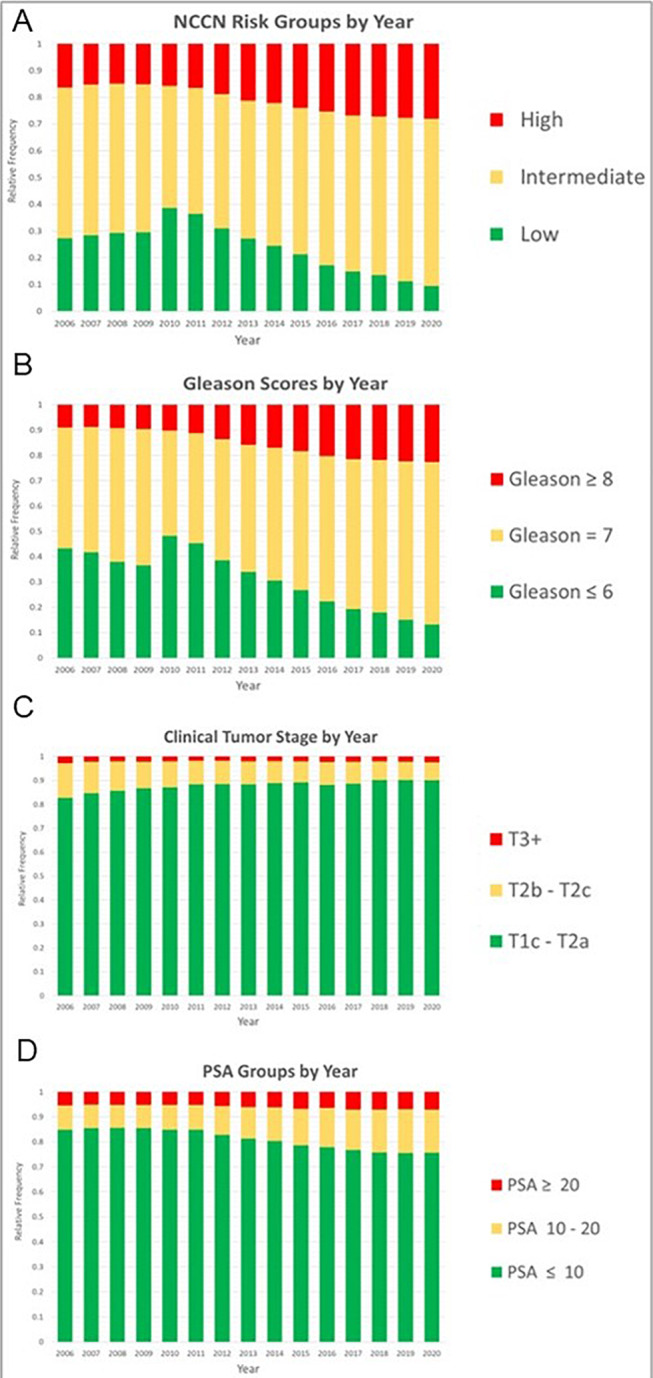
Annual proportions of (**A**) NCCN risk Groups; (**B**) Biopsy Gleason scores; (**C**) Clinical tumor stage; and (**D**) PSA categories

Over the three time periods, the percentages of high-risk patients were 15%, 20%, and 27%, while intermediate risk were 54%, 51%, and 60%, respectively. As shown in Fig. 2A, these shifts corresponded with a 58% drop in low-risk cases (*p* < 0.001 for all). The analysis of individual components of the NCCN risk classification reveals an increase in GS ≥ 8 from 9 to 22%, and GS 7 from 47 to 64% between 2006 and 2020 (*p* < 0.001). During the same period, levels of PSA ranging from 10–20ng/dL increased from 10 to 17%, while no clinically significant changes occurred in cT3 + stage and PSA > 20 ng/mL categories. Correspondingly, there were reductions in the percentages of low-risk features, specifically in GS 6 and PSA levels < 10 ng/dL. These temporal trends are illustrated in Fig. 2b and d.

**Fig. 3:**
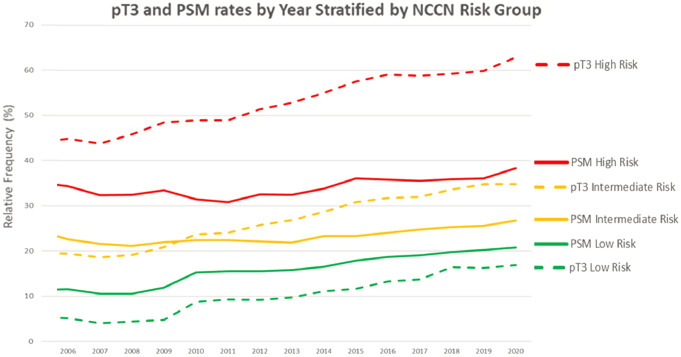
Rates of pT3 and positive surgical margins (PSM) according to NCCN risk groups by year

**Fig. 4:**
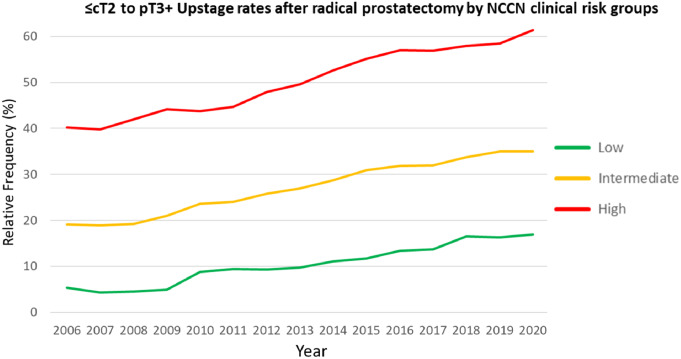
Tumor upstaging rates after radical prostatectomy according to NCCN risk groups

Overall, pT3 rates rose from 20 to 38%, and PSM rates from 20 to 27% across the studied time periods (*p* < 0.001 for both). Elevations in both pT3 rates and PSM were significant across all NCCN risk groups, although growth rates differed among them (Fig. 3). Considering the three time periods, PSM rates were 15%, 16%, and 18% for pT2 (*p* < 0.001), and 42%, 41%, and 43% for pT3 cases (*p* = 0.0164). Similarly, rising tumor upstaging rates were observed in low (6–15%), intermediate (20–33%), and high-risk patients (42–58%) between the first and last time periods (*p* < 0.001 for all, Fig. 4).

**Fig. 5:**
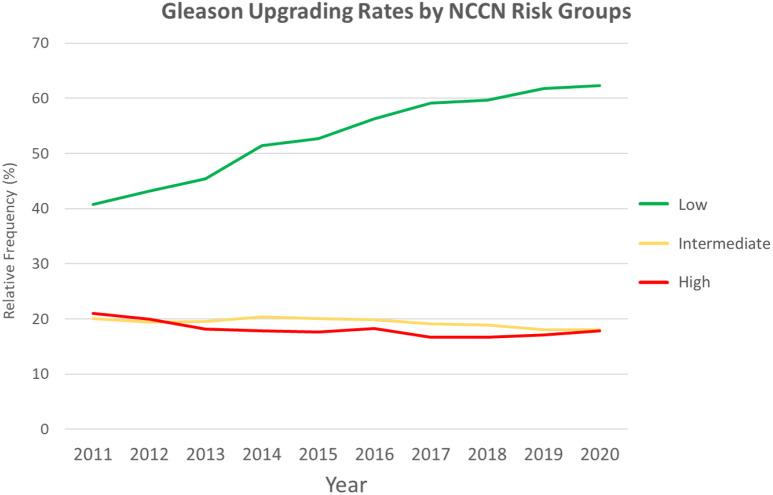
Gleason Group (GG) upstaging rates after radical prostatectomy according to NCCN risk groups

Gleason group upgrading showed different trends according to NCCN risk groups. As illustrated in Fig. 5, upgrading rates in the low-risk group rose from 45% in 2011–2015 to 59% in 2016–2020 (*p* < 0.001). Conversely, intermediate and high-risk patients demonstrated slight reductions, respectively, from 20 to 19% (*p* < 0.001), and 19–17% in the same time periods (*p* < 0.001).

## Discussion

Our study shows a trend towards the surgical treatment of more advanced PCa, in particular, due to higher grade disease, more lymph node metastases, and rising rates of PSM and pT3 across all NCCN risk groups. Radical prostatectomy has been performed in slightly older men with more comorbidities. A higher representation of African American patients was observed, likely as a result of selection bias due to their higher rates of aggressive disease; yet other competing factors (e.g. disparities in access to health care) cannot be ruled out. We also observed a notable and steady rise in pathologic upstaging across all risk groups after radical prostatectomy between 2006 and 2020.

The elevation in pT3 rates is likely multifactorial: in addition to the growing utilization of RP for treating high-risk PCa, more low and favorable intermediate-risk cases have been managed with active surveillance or focal therapy, rather than immediate surgery [13, 14]. Since 2021, AS has become the preferred treatment modality according to the NCCN guidelines for very low and low-risk PCa. Moreover, the shift towards less intense screening strategies might delay diagnosis and result in more advanced disease stages at the time of radical prostatectomy [8, 15]. The increase in pT3 rates within intermediate and especially low-risk categories suggests a shift towards higher-risk PCa among men undergoing surgery, both across and within risk groups. In fact, contemporary low-risk patients undergoing RP likely have more adverse features than in the past. These factors might also have contributed to the more than two-fold increase in pathologic node-positive disease over the last 15 years. As a result, fewer patients might be cured with surgery alone, necessitating more multimodal therapy, which adversely impacts functional outcomes.

The notable increase in tumor pathologic upstaging across all NCCN groups is primarily a consequence of the growing discrepancy between stable rates of clinical and rising pathologic T3 stages. This trend persisted despite greater availability and refined expertise with prostate MRI [16]. This observation may reflect MRI’s limited sensitivity in detecting extraprostatic extension [17]. Additionally, persistent use of DRE alone for clinical staging may understage cases with limited extraprostatic extension. Still, such observations merit further investigation as they raise concern for unanticipated upstaging in this population, which is associated with lower rates of Pentafecta (functional, oncologic, and perioperative success) [18].

Gleason upgrading exhibited differing trends across NCCN risk groups that reflect the evolution of PCa management in the last years. The significant elevation in upgrading among low-risk cases suggests a more selective indication of RP for those with unfavorable features within this category, such as high-volume Gleason 6 or elevated PSA density. Also, Gleason 6 (Grade Group 1) tumors are less visible in multiparametric MRI. In other words, trends in NCCN low-risk group indicate a more selective application of surgery to cases in the highest spectrum of risk. Conversely, the minimal reduction in upgrading rates in intermediate and high-risk patients is relevant as they would be expected to rise amidst the major shift to higher-risk disease observed in RPs. This suggests an improved detection of the highest-grade disease within the prostate before surgery, likely due to the expansion of imaging and targeted biopsies. This hypothesis is supported by evidence demonstrating that multiparametric MRI not only improves the detection of clinically significant PCa [19]but also reduces pathologic upstaging in the RP specimen [20].

Rising PSM rates have been previously reported and largely mirror the significant increase in pT3 rates across all risk groups [8, 10]. PSM increased despite the growing adoption and accumulated experience with the robotic platform over the years. Combined with enhanced precision and magnified visualization, one would expect stable or even declining PSM rates, particularly for organ-confined (pT2) disease. Thus, the observed 20% increase in PSM rates in pT2 cases is concerning for surgical decision-making because more conservative approaches, such as fascia and nerve-sparing techniques, might increase the incidence and extension of PSM, potentially risking RP oncological effectiveness. As surgeons may choose to perform less fascia and nerve-sparing procedures, our findings carry implications on functional outcomes as well. Indeed, this was highlighted by a single-center study of 10,000 RPs which showed a decrease in nerve-sparing procedures as pT3 and PSM rates went up [8]. Consequently, the current rates of continence and potency may no longer be comparable to the early robot-assisted RP series [9, 21, 22]. Although still frequently cited as reference standards for functional outcomes, these series contain higher proportions of low-risk and favorable cases. Therefore, our findings suggest that surgical protocols and patient counseling may need to be revised to more accurately reflect these recent changes.

The strengths of our study lie in its detailed information on demographics, cancer features, and postoperative outcomes coupled with a large hospital-based cohort. This allows for a broader understanding of how the RP population has changed over time. While not a population-based registry, NCDB captures more than 70% of cancers diagnosed and treated annually in the United States across more than 1,500 commission on cancer-accredited facilities [12, 23]. This makes it particularly valuable for assessing real-world practices, in contrast to previous studies addressing changes in RP patient profiles from academic centers, known for achieving superior outcomes [24].

The main limitations of our study are linked to the retrospective analysis of large datasets, which inherently includes risks of bias. However, the NCDB is a substantial national dataset that is well-suited for analyzing trends. Another limitation is the 2005 ISUP major review of the Gleason grading system, which experienced a delay in adoption. Consequently, Gleason scores, particularly from the initial years following this review, may not be entirely comparable even after limiting analysis to the period post-modifications [25]. Lastly, NCDB does not capture MRI utilization and whether clinical tumor stage was based on digital rectal examination (DRE) or MRI findings. As MRI has superior local staging accuracy [26], persistent utilization of DRE can lead to rising pathologic upstaging rates particularly when the population is shifting to higher-risk disease.

## Conclusions

Contemporary data from a large hospital-based registry indicate that RP is increasingly performed on men with more comorbidities and higher risk PCa. This trend is marked by rising rates of PSM, pT3, and pathologic upstaging across all NCCN risk groups, despite growing experience with prostate MRI and robotic surgery. Gleason upgrading rates suggest a more selective indication for RP in low-risk cases and improved detection of high-grade cancer in the prostate before surgery, likely due to the expansion of targeted biopsies. These trends should guide surgical decision-making, emphasizing the need for a careful balance between the use of fascia and nerve-sparing techniques to avoid compromising oncological safety.

## Data Availability

This study used data made available by the National Cancer Database (NCDB) through the Participant User Data File (PUF), a HIPAA-compliant, de-identified dataset. The PUF includes patient-level data submitted to the NCDB by Commission on Cancer (CoC)-accredited programs. Access to NCDB PUFs is restricted to participating programs and requires an application process. Therefore, the original dataset cannot be disclosed.
